# The Inventory of Physical Activity Barriers for Adults 50 Years and Older: Refinement and Validation

**DOI:** 10.1093/geroni/igab046.2894

**Published:** 2021-12-17

**Authors:** Mariana Wingood, Salene Jones, Nancy Gell, Denise Peters, Jennifer Brach

**Affiliations:** 1 University of Vermont, University of Vermont, Vermont, United States; 2 Fred Hutchinson Cancer Research Center, Seattle, Washington, United States; 3 University of Vermont, Burlington, Vermont, United States; 4 University of pittsburgh, Pittsburgh, Pennsylvania, United States

## Abstract

Addressing physical activity (PA) barriers is an essential component of increasing PA among the 56-73% of community-dwelling adults 50 years and older who are not performing the recommended 150 minutes of moderate-to-vigorous PA. As there is no feasible, multi-factorial tool to assess PA barriers among this population, we developed and validated a PA barrier assessment tool called the Inventory of Physical Activity Barriers (IPAB). We collected cross-sectional data on 503 adults (mean age 70.1), with 79 participants completing the scale twice for test-retest reliability and 64 completing a cross-over design examining the ability to use two administration formats interchangeably. Our analyses consisted of exploratory and confirmatory factor analysis, Cronbach alpha, intraclass correlation coefficient, Bland-Altman Plot, and t-tests. Using factor analysis, we identified and confirmed an eight-factor solution consisting of 27 items. The 27-item IPAB is internally consistent (alpha= 0.91), has a high test-retest reliability (intraclass correlation coefficient=0.99), and can differentiate between individuals who meet the recommended levels of PA and those who do not (p < 0.001). The IPAB scores ranged between 1.00-3.11 for the paper format (mean=1.78) and 1.07-3.48 for the electronic format (mean=1.78), with no statistical difference between the paper and electronic administration formats (p=0.94), resulting in the conclusion that the two administration formats can be used interchangeably. Participant feedback illustrates that the IPAB is easy to use, has clear instruction, and is an appropriate length. The newly validated IPAB scale can be used to develop individualized PA interventions that address PA barriers among patients 50 years and older.

